# Zinc finger C3H1 domain-containing protein (ZFC3H1) evaluates the prognosis and treatment of prostate adenocarcinoma (PRAD): A study based on TCGA data

**DOI:** 10.1080/21655979.2021.1965442

**Published:** 2021-09-13

**Authors:** Hang Huang, Haokai Xu, Ping Li, Xueting Ye, Wei Chen, Wei Chen, Xixi Huang

**Affiliations:** a Department Of Urology, The First Affiliated Hospital of Wenzhou Medical University, Wenzhou, China; b Department Of Surgery, Ningbo, Zhejiang, China; c Cancer Institute Of Integrated Traditional Chinese And Western Medicine, Key Laboratory Of Cancer Prevention And Therapy Combining Traditional Chinese And Western Medicine, Zhejiang Academy Of Traditional Chinese Medicine, Hangzhou, Zhejiang, China

**Keywords:** ZFC3H1, prostate adenocarcinoma, computational biology, cell migration, cell invasion, prognosis

## Abstract

The present study was aimed to evaluate the expression profile of Zinc finger C3H1 domain-containing protein (ZFC3H1) using bioinformatic analysis of public datasets from The Cancer Genome Atlas database (TCGA). The results showed that the expression levels of ZFC3H1 were notably lower than the corresponding non-cancerous tissues in prostate adenocarcinoma (PRAD), and patients in the high ZFC3H1-expression group showed poor survival. We hypothesized that the low expression of ZFC3H1 in tumor tissue might have be an inhibitory effect on the autoimmune system. We predicted the regulatory target and protein interaction partner network of ZFC3H1, and identified a PPI network composed of 26 node genes in PRAD. Furthermore, we found that the expression levels of *MPHOSPH6* (encoding M-phase phosphoprotein 6) and *MRPS31* (encoding mitochondrial ribosomal protein S31) were lower in PRAD tissues than in non-cancerous tissues, and the survival time of patients with high MPHOSPH6 and MRPS31 expression was poor. To further demonstrate the role of ZC3H1 in PRAD, we knocked-down the *ZFC3H1* expression and found that the inhibition of *ZFC3H1* significantly inhibited PRAD cell migration and invasion. Furthermore, *ZFC3H1 siRNA* treatment could reduce cell viability and increase the number of apoptotic cells in PRAD cells. Taken together, ZFC3H1 could represent a new marker for PRAD prognosis and provide a reference for the development of new therapies to treat PRAD.

## Introduction

Cancer is the second most common cause of death worldwide (8.97 million deaths) after ischemic heart disease [1]. PRAD is the second most commonly diagnosed cancer and the second leading cause of cancer mortality in men [2]. The 5-year survival rate of PRAD is about 70–100%; however, for patients with distant metastatic cancer, the 5-year survival rate is only 30% [3]. Among the most prevalent solid tumors with advanced disease, PRAD has the fewest therapeutic options, and advanced PRAD remains incurable [4,5]. Therefore, accurately evaluating the prognosis of PRAD is vital.

Zinc finger C3H1 domain-containing protein (ZFC3H1), also designated as CCDC131 (coiled-coil domain containing protein), or PSRC2 (proline/serine-rich coiled-coil protein), is a large protein (1989 amino acids), with a C3H1- type zinc finger motif in its center, and contains five tetratricopeptide (TRP) repeats and six half-tetratricopeptide (HAT) repeats at the C-terminal region. ZFC3H1 is a central factor in the retention and degradation of polyadenylated RNA, and is involved in the processing of a wide range of RNAs, playing a crucial role in the degradation of nuclear RNAs [6–9]. Garland et al. reported a disruptive relationship between excess RNA and polycomb repressive complex 2 (PRC2) upon depletion of ZFC3H1 in mouse ESCs [10]. In addition, there are also reports that the depletion of ZFC3H1 resulted in a significant inhibition of translation [11]. The reason may be that ZFC3H1 plays an important role in promoting turnover of unstable nuclear RNAs and preventing their cytoplasmic transport and global translational repression. When its activity decreases, normally unstable RNAs accumulate and are transported to the cytoplasm, thus inhibiting translation globally [12]. ZFC3H1 is also reported to play an important role in regulating cytokine production and RNA decay [13–15]. Considering that advanced PRAD depends on protein synthesis to maintain its survival and accelerate metabolism to promote growth [16], we hypothesized that ZFC3H1 would correlate with the prognosis of PRAD and play an active role in the progression of PRAD.

In the present study, based on The Cancer Genome Atlas (TCGA) database, we aimed to analyze the expression levels of *ZFC3H1* and its target genes in PRAD, explore the role of ZFC3H1 in PRAD, and finally determine whether ZFC3H1 could provide a reference to evaluate PRAD prognosis and treatment by detecting its influence on the migration and invasion of PRAD cells.

## Methods

### ZFC3H1 expression in PRAD samples from the TCGA database

The TCGA is a central repository of multidimensional experimental cancer data, comprised of data pertaining to more than 30 types of human tumors. We obtained the expression profile of *ZFC3H1* in different human cancers and corresponding non-cancerous tissues (Table 1), including PRAD, as well as its correlation with the prognosis of patients with PRAD, via the Gene Expression Profiling Interactive Analysis (GEPIA) tool (http://gepia.cancer-pku.cn/), which is based on the TCGA database [17]. Entering ZFC3H1 in the ‘General’ field and clicking the “’GoPIA!’: GEPIA generated the expression profile of ZFC3H1.

**Table 1.** TCGA datasets evaluated (data from TCGA datasets)

Types of cancer	TCGA dataset	No. of cancer tissues	No. of normal tissues
Adrenocortical carcinoma	TCGA-ACC	77	128
Bladder Urothelial Carcinoma	TCGA-BLCA	404	28
Breast invasive carcinoma	TCGA-BRCA	1085	291
Cervical squamous cell carcinoma and endocervical adenocarcinoma	TCGA-CESC	306	13
Cholangio carcinoma	TCGA-CHOL	36	9
Colon adenocarcinoma	TCGA-COAD	275	349
Lymphoid Neoplasm Diffuse Large B-cell Lymphoma	TCGA-DLBC	47	337
Esophageal carcinoma	TCGA-ESCA	182	286
Glioblastoma multiforme	TCGA-GBM	163	207
Head and Neck squamous cell carcinoma	TCGA-HNSC	519	44
Kidney Chromophobe	TCGA-KICH	66	53
Kidney renal clear cell carcinoma	TCGA-KIRC	523	100
Kidney renal papillary cell carcinoma	TCGA-KIRP	286	60
Acute Myeloid Leukemia	TCGA-LAML	173	70
Brain Lower Grade Glioma	TCGA-LGG	518	207
Liver hepatocellular carcinoma	TCGA-LIHC	369	160
Lung adenocarcinoma	TCGA-LUAD	483	347
Lung squamous cell carcinoma	TCGA-LUSC	486	338
Mesothelioma	TCGA-MESO	87	0
Ovarian serous cystadenocarcinoma	TCGA-OV	426	88
Pancreatic adenocarcinoma	TCGA-PAAD	179	171
Pheochromocytoma and Paraganglioma	TCGA-PCPG	182	3
Prostate adenocarcinoma	TCGA-PRAD	492	152
Rectum adenocarcinoma	TCGA-PEAD	92	318
Sarcoma	TCGA-SARC	262	2
Skin Cutaneous Melanoma	TCGA-SKCM	461	558
Stomach adenocarcinoma	TCGA-STAD	408	211
Testicular Germ Cell Tumors	TCGA-TGCT	137	165
Thyroid carcinoma	TCGA-THCA	512	337
Thymoma	TCGA-THYM	118	339
Uterine Corpus Endometrial Carcinoma	TCGA-UCEC	174	91
Uterine Carcinosarcoma	TCGA-UCS	57	78

### Prediction and screening of target genes

The target genes of ZFC3H1 were predicted using three databases: STRING (https://string-db.org/), BioGRID (https://thebiogrid.org/), and IntAct (https://www.ebi.ac.uk/intact/) [18–20]. To improve the accuracy of the prediction results and to construct Venn diagrams, we chose target genes that overlapped between at least two of the three databases (http://bioinformatics.psb.ugent.be/webtools/Venn/).

### Enrichment analysis of overlapping target genes in the ZFC3H1 signaling pathway

Enrichment analysis of overlapping target genes in the ZFC3H1 signaling pathway, was performed using Metascape (http://metascape.org/gp/index.html)[21]. We analyzed the function of these genes in tumor biological processes via gene ontology (GO) enrichment analysis [22].

### Identifying node degree genes via protein–protein interaction (PPI) network analyses of overlapping target genes

PPI analysis was performed using overlapping target genes of ZFC3H1 through Metascape, which identified 26 node degree genes [21]. Molecular Complex Detection (MCODE) algorithm, a module in Cytoscape v1.1 [23] was used to identify densely connected network neighborhoods. Each MCODE component was labeled with a different color, and their biological significance was characterized.

### Prognostic significance of the chosen node degree genes

To prevent any errors resulting from the use of a single database, the 26 node degree genes were analyzed using the starbase (http://starbase.sysu.edu.cn/index.php) tool to observe whether their expression levels varied between PRAD and corresponding non-cancerous tissues, and whether these genes had any influence on the prognosis of PRAD [24]. The gene symbol or gene ID (Ensembl ID) of node genes was entered in the ‘Expression DIY’ and ‘Survival Analysis’ fields respectively, and PAAD selected in the ‘Dataset’ field. Clicking the ‘Plot’ button caused GEPIA to present the gene expression box plot and survival plot of ZFC3H1 in PAAD respectively.

### Cell culture

Human PRAD cells (22RV1, DU145) were obtained from American Type Culture Collection (Manassas, VA, USA). 22RV1 and DU145 cells were maintained in Roswell Park Memorial Institute (RPMI) 1640 medium (Gibco, Grand Island, NY, USA), supplemented with 10% fetal bovine serum (FBS; Gibco) and 1% penicillin/streptomycin (Sigma, St. Louis, MO, USA). All cells were incubated at 37°C in a humidified atmosphere containing 5% CO_2_.

### RNA transfection

Small interfering RNAs (siRNAs) against ZFC3H1, along with RNAi negative controls, were synthesized by Genepharm Technologies (GenePharma Ltd., Shanghai, China). The PRAD cells’ transfection was performed by/using Lipofectamine 2000 (Invitrogen Corporation, Carlsbad, CA, USA) according to the manufacturer’s protocol. The siRNA sequences were as follows: ZFC3H1-homo-2234: 5´- CCAAGAAGCAAUCUAUCAATT-3´ and 5´-UUGAUAGAUUGCUUCUUGGTT-3´; ZFC3H1-homo-3986: 5´- GGAGUAAACAAAGAUCGAATT-3´ and 5´- UUCGAUCUUUGUUUACUCCTT-3´; ZFC3H1-homo-5975: 5´-GCUGCUGAGAUUGUUCUAATT-3´ and 5´- UUAGAACAAUCUCAGCAGCTT −3´; Negative control: 5´-UUCUCCGAACGUGUCACGUTT-3´ and 5´-ACGUGACACGUUCGGAGAATT-3´.

### Wound healing assay

Cell migration ability was measured by the wound healing assay as described. For transfection with ZFC3H1 siRNA or negative siRNA, cells were plated in six-well plates at 3 × 10^5^ cells/well. A 200-μl micropipette tip was used to create a wound scratch across a confluent monolayer of cells. After saline washing, capsules were visualized at 0 and 24 h using an inverted light microscope (Olympus IX51, Center Valley, PA, USA). Wound closure rate ((initial wound area – wound area at 24 h)/initial wound area) was calculated, and the wound area was quantified using ImagePro Plus v. 6.0 (Media Cybernetics, Bethesda, MD, USA).

### Transwell invasion assay

The cell invasion assay was conducted using 24-well transwell chambers (pore size 8 μm; Corning Inc., Corning, NY, USA). For invasion assays, the upper chambers of inserts were coated with 70 μl of Matrigel (1 mg/ml, BD Bioscience, San Jose, CA, USA). Transfected PRAD cells (5 × 10^4^) in a serum-free medium, were seeded in the top chamber, while 700 μL of medium containing 10% FBS was added to the lower chamber. After a 24 h incubation, the cells were fixed with methanol for 10 min, and stained with 1% crystal violet at room temperature for 10 min. The number of migrated cells was counted and photographed in five randomly selected fields under an inverted microscope (×40 magnification; Olympus Corporation, Tokyo, Japan).

### Western blotting

Total cell proteins were extracted using 1× cell lysis buffer (Cell Signaling Technology Inc., Danvers, MA, USA), after which the protein concentration was quantified using a Bicinchoninic Acid (BCA) kit (Sigma-Aldrich; Merck KGa). 40 μg of proteins in each sample were separated by 10% sodium dodecyl sulfate-polyacrylamide gel electrophoresis (SDS-PAGE) and transferred onto polyvinylidene difluoride (PVDF) membranes (EMD Millipore, Billerica, MA, USA). After the transfer, the membranes were blocked with 5% nonfat milk in Tris-buffered saline, and then incubated with the primary antibody against E-cadherin, vimentin, and Zinc finger C3H1 domain-containing protein (ZFC3H1) (Abcam, Cambridge, MA, USA), diluted 1:1000 in TBS-T overnight at 4°C. After washing them in TBS-T three times, with 10 min intervals, the membranes were incubated with the appropriate horseradish peroxidase–labeled secondary antibody (Abcam, Cambridge, MA, USA), diluted 1:2000 in TBS-T for 1.5 h at room temperature. Glyceraldehyde-3-phosphate dehydrogenase (GAPDH) protein expression was used as the internal control. Protein signals were detected using enhanced chemiluminescence reagents (Pierce Biotechnology, Inc., Rockford, IL, USA) and the immunoreactive protein’s final signals were detected by Image Lab Software Version 5.0 (Bio-Rad, Hercules, CA, USA).

### qRT-PCR detection

Total RNA was extracted from 22RV1 cells and DU145 cells by TRIzol reagent (Invitrogen), and the primers were synthesized by Genepharm Technologies (GenePharma Ltd., Shanghai, China). The target gene and internal reference gene of each sample were amplified simultaneously. 2^−∆∆Ct^ was applied to analyze the data. Primer sequence of ZFC3H1: forward primer: 5´-TGTCTCAGTGTCATACCCATCT-3´, reverse primer: 5´-CCTTCTGGGGTCTGAAAGAACTT-3´. Primer sequence of GADPH: forward primer: 5´-AGAAGGCTGGGGCTCATTTG-3´, reverse primer: 5´-AGGGGCCATCCACAGTCTTC-3´.

### CCK-8 assay

After 48 h of transfection, cells were collected and detected by CCK-8 kit (CCK-8; Dojindo, Japan). The cell density of 22RV1 and DU145 was adjusted to 5 × 10^3^ cells/well, planted in a 96-well plate. The CCK-8 reagent was added to each well for 2 h, respectively, and then the cells were cultured at 37°C for another 1 h.

### Flow cytometry analysis

According to the manufacturer’s protocol, we used the Annexin V-FITC cell apoptosis detection kit to determine the number of apoptotic cells. Briefly, the PRAD cells were transfected with ZFC3H1 siRNA later, prepared with a single-cell suspension using 2 × 10^5^ cells. Then, the supernatant was discarded in the dark, and a 5 μl/sample of FITC-labeled Annexin-V was added and incubated for 30 min. Following this, a 5 μl/sample of PI was added and allowed to react for 5 min. The number of apoptotic cells was counted using flow cytometry.

### Immunohistochemical staining

Immediately after harvesting PRAD tissues, they were rinsed in PBS, fixed in buffered paraformaldehyde solution (4%) and embedded in paraffin. After cutting paraffin samples of PRAD tissues into 3 μm thick slices, they were dried in an oven at 60°C for 1 h, then dewaxed twice in xylene, rinsed with ethanol, and rehydrated in alcohol solutions with decreasing gradient. Paraffin sections were incubated with 2% H2O2 for 15 min after heat-induced antigen retrieval in a 10 mM citrate buffer (pH 6.0), then washed 3 times and incubated in 0.3% Triton X-100 PBS for 2 h. The paraffin sections were then blocked by incubation with 5% BSA for 1 h. The slides were incubated with an anti–ZFC3H1 antibody (1:500, Abcam), overnight at 4°C. The corresponding second antibody incubated for 20 min at 37°C and a diaminobenzidine (DAB) DAB substrate kit (Abcam) were used as detection reagents. At last, the sections were dehydrated, mounted and observed under light microscopy (Olympus, Tokyo, Japan). The positive rates were measured using Image-Pro Plus v. 6.0 software (Media Cybernetics, Bethesda, MD, USA).

## Results

### ZFC3H1 expression in PRAD samples from the TCGA database

**Figure 1.**
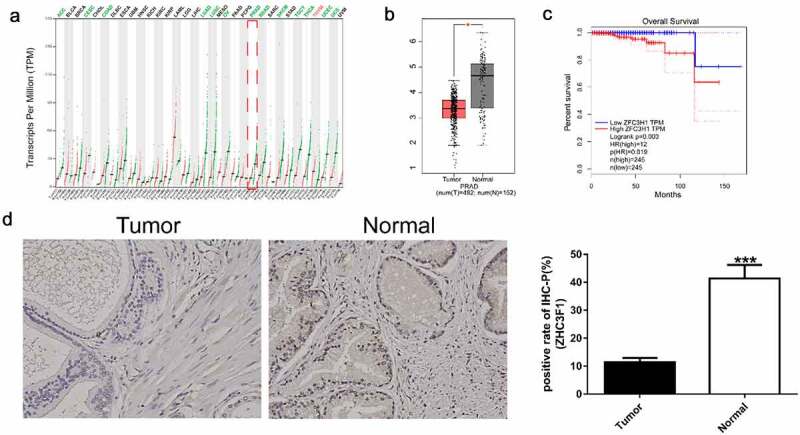
The correlation between *ZFC3H1* expression levels and overall survival of patients with PRAD. A, *ZFC3H1* expression profile across all tumor samples and paired normal tissues. B, The expression level of ZFC3H1 in PRAD tissues and adjacent non-tumor tissues. C, The overall survival of human PRAD patients in relation with high or low expression levels of ZFC3H1. **D. Immunohistochemical staining examined the expression of ZFC3H1 in 10 prostate cancer specimens and paracancerous.**

As shown in Figure 1a and Figure 1b, the expression of ZFC3H1 was low in many human cancers, such as PRAD, adrenocortical carcinoma, cervical squamous cell carcinoma and endocervical adenocarcinoma, colon adenocarcinoma, lung adenocarcinoma, lung squamous cell carcinoma, ovarian serous cystadenocarcinoma, rectum adenocarcinoma, skin cutaneous melanoma, testicular germ cell tumors, thyroid carcinoma, uterine corpus endometrial carcinoma, and uterine carcinosarcoma, but showed high expression only in thymoma. Unexpectedly, however, as shown in Figure 1c, the survival time of patients with PRAD and high expression of ZFC3H1 was poor. We also detected ZFC3H1 expression in 10 prostate cancer specimens and paracancerous, by immunohistochemical staining which indicated that the ZFC3H1 expression was down-regulated in tumors when compared with Normal (Figure 1 D). These results indicate that ZFC3H1 might enhance the progression of PRAD.

### Prediction and screening of target genes

**Figure 2.**
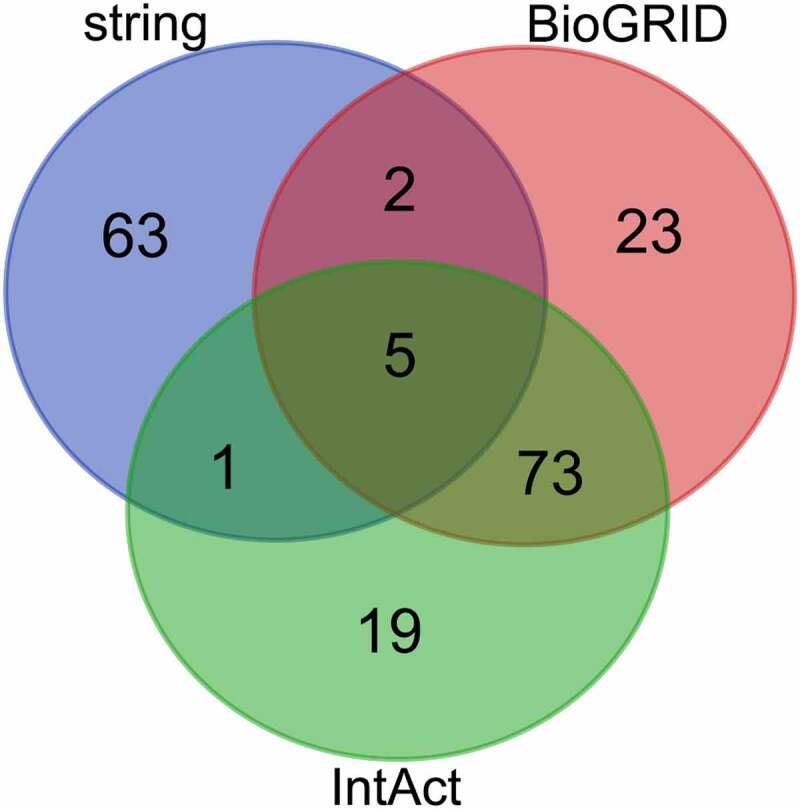
Venn diagram of predicted target genes from three databases

Analysis using the STRING, BioGRID, and IntAct databases, led to the identification of 71, 103, and 98 genes, respectively (Table 2). We selected genes that overlapped between at least two of the three databases to be the target genes, which produced a shortlist of 81 potential target genes of ZFC3H1. A Venn diagram was then constructed for these target genes (Figure 2).

**Table 2.** Prognosis adenocarcinoma-associated genes identified in The cancer genome atlas using four databases (data from TCGA datasets)

Database	Gene name
STRING	*BMS1C1DCDC5LDCAF13DCP2DDX47DDX49DDX52DIS3DIS3LEMG1EXOSC1EXOSC10EXOSC2EXOSC3EXOSC4EXOSC5EXOSC6EXOSC7EXOSC8EXOSC9FBLHBS1LHEATR1HNRNPDIMP3IMP4KHSRPKRR1MPHOSPH6MPP6NOC4LNOL6NOP14NOP56PDCD11PNPT1PWP2RCC1RPL23ARPL27ARPL30RPL32RPL38RPL39RPL5RRP7ARRP9SKIV2LSKIV2L2SUPV3L1TBL3TDP2TEX10TTC37UTP11 LUTP14AUTP14BUTP15UTP18UTP3UTP6WDR3WDR36WDR43WDR46WDR61WDR75XRN1ZFC3H1ZFP36*
BioGRID	*ACTR1AADARANGEL2ANKHD1-EIF4EBP3ANKRD52APPBUB1BBUB3C1DC9ORF41CCDC57CCDC85BCEP70CSNK2A1CUL7DAP3DDB1DGKZDHX30DOCK8EIF2B1ERCC1ERCC4ESR2EXOSC1EXOSC5EXOSC7FBXO38FBXW8GANGOLGA2GPN3HIST1H4AHNRNPCL1HNRNPKHTTKHDRBS2KIAA1429LARP1LARP1BLENG8LGR4MAPK9MCATMIB1MKRN2MOV10MPHOSPH6MRPS11MRPS14MRPS23MRPS24MRPS25MRPS27MRPS31MRPS35MRPS5MRPS7MRPS9MTERF3MTUS2MYH13NINLNOL6PABPC4PAPOLGPCID2PPHLN1PPP1CCPPP2CAPPP2R2DPPP2R3APRMT6PTCD3RALYRBM27RBM45RNF4RPAP2SKIV2L2SLBPSMYD2SPAG5SRGNSRPK2SRRTSUFUTACC3TBC1D1TBCETHOC1TP53BP1TRIM27UNC119BUPF1VPRBPVPS26BVPS72YLPM1ZC3H18ZC3H3ZCCHC3ZDHHC17*
IntAct	*ACTR1AADARANGEL2ANKHD1APPBUB1BBUB3C1DCADPSCARNMT1CCDC57CCDC85BCDC5LCEP70COG6CSNK2A1CUL7DCAF1Ddb1DHX30DOCK8EIF2B1ERCC1ERCC4ESR1EXOSC1EXOSC7FBXW8FMR1GOLGA2GPN3H1-2H1-4H1-5HNRNPCL1HNRNPKHSF2BPHTTKHDRBS2LARP1LENG8MAPK9MKRN2MKRN3MOV10MPHOSPH6MRPS11MRPS14MRPS23MRPS24MRPS25MRPS31MRPS35MRPS5MRPS7MTERF3MTREXMTUS2MYBMYH13NINLNOL6NS1PABPC4PAPOLGPCID2PIBF1PPHLN1PPP2CAPPP2R2DPPP2R3APRMT6PTCD3q7ard3_yerpeRALYRBM27RBM45RPAP2RPL36SLBPSMYD2SPAG5SRGNSRRTSUFUTACC3TBC1D1TBCETHOC1TNIKTP53BP1TRIM27UNC119BUPF1VPS26BZC3H18ZC3H3ZDHHC17*

### Enrichment analysis of overlapping target genes in the ZFC3H1 signaling pathway

GO enrichment analyses was performed to determine the functions of the 81 target genes in tumor biological processes (Table 3). The putative target genes of ZFC3H1 were found to be enriched in certain cell processes, such as translational termination, regulation of the mRNA metabolic process, mRNA processing, mitotic nuclear division, mRNA surveillance pathway, cell cycle phase transition, RNA 3ʹ-end processing, positive regulation of the catabolic process, nucleic acid phosphodiester bond hydrolysis, nucleic acid transport, ribosome assembly, microtubule cytoskeleton organization, etc.

**Table 3.** Pathway enrichment analysis of overlapping target genes (data from TCGA datasets)

Term	Description	Count	Frequency, %	Log_10_(P)	Log_10_(q)
GO:0006415	translational termination	11	13.75	−14.02	−9.86
GO:1,903,311	regulation of mRNA metabolic process	12	15	−9.73	−6.55
GO:0006397	mRNA processing	13	16.25	−8.34	−5.36
GO:0140014	mitotic nuclear division	9	11.25	−6.73	−3.87
ko03015	mRNA surveillance pathway	6	7.5	−6.62	−3.8
GO:0044770	cell cycle phase transition	12	15	−6.59	−3.78
GO:0031123	RNA 3ʹ-end processing	7	8.75	−6.56	−3.76
GO:0009896	positive regulation of catabolic process	10	12.5	−6.22	−3.49
GO:0090305	nucleic acid phosphodiester bond hydrolysis	8	10	−5.58	−3
GO:0050657	nucleic acid transport	6	7.5	−4.66	−2.19
GO:0042255	ribosome assembly	4	5	−4.47	−2.06
GO:0000226	microtubule cytoskeleton organization	9	11.25	−4.23	−1.88
GO:0045070	positive regulation of viral genome replication	3	3.75	−4.03	−1.72
GO:0043632	modification-dependent macromolecule catabolic process	9	11.25	−3.98	−1.68
GO:0044089	positive regulation of cellular component biogenesis	8	10	−3.89	−1.6
GO:0006914	autophagy	8	10	−3.81	−1.54
GO:0042769	DNA damage response, detection of DNA damage	3	3.75	−3.73	−1.5
GO:0031330	negative regulation of cellular catabolic process	5	6.25	−3.08	−0.99
GO:1,901,796	regulation of signal transduction by p53 class mediator	4	5	−2.78	−0.76
GO:0030099	myeloid cell differentiation	5	6.25	−2.15	−0.28
GO:0044839	Cell cycle G2/M phase transition	11	47.83	−15.46	−11.14
GO:0042752	Regulation of circadian rhythm	7	30.43	−10.97	−7.66
GO:0045786	Negative regulation of cell cycle	9	39.13	−8.47	−5.51
GO:0097193	Intrinsic apoptotic signaling pathway	6	26.09	−6.63	−3.91
GO:0031648	Protein destabilization	3	13.04	−4.84	−2.67

### Identifying node degree genes via PPI network analyses of overlapping target genes

**Figure 3.**
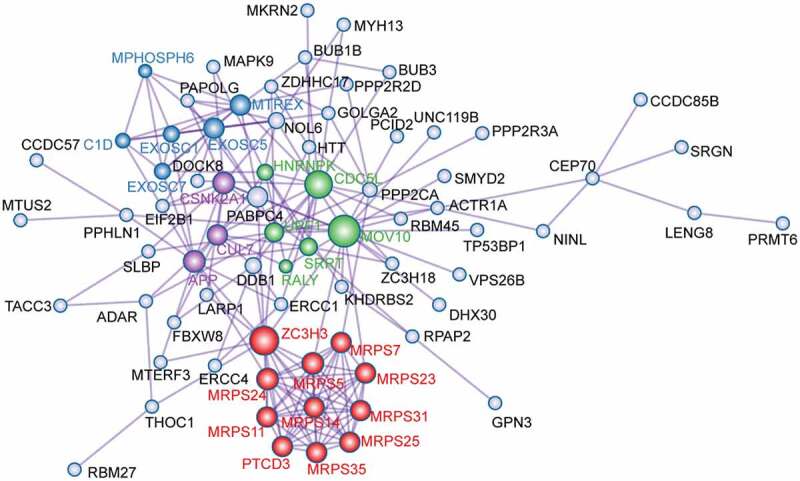
Protein-protein interaction network of overlapping target genes

To explore the interaction between the 81 overlapping target genes, a PPI network was constructed using Metascape (Figure 3). From the PPI network, 26 node degree genes were identified: *APP, C1D, CDC5L, CSNK2A1, CUL7, EXOSC1, EXOSC5, EXOSC7, HNRNPK, MOV10, MPHOSPH6, MRPS11, MRPS14, MRPS23, MRPS24, MRPS25, MRPS31, MRPS35, MRPS5, MRPS7, MTREX, PTCD3, RALY, SRRT, UPF1*, and *ZC3H3*.

### Prognostic significance evaluation of the twenty-six node degree genes

**Figure 4.**
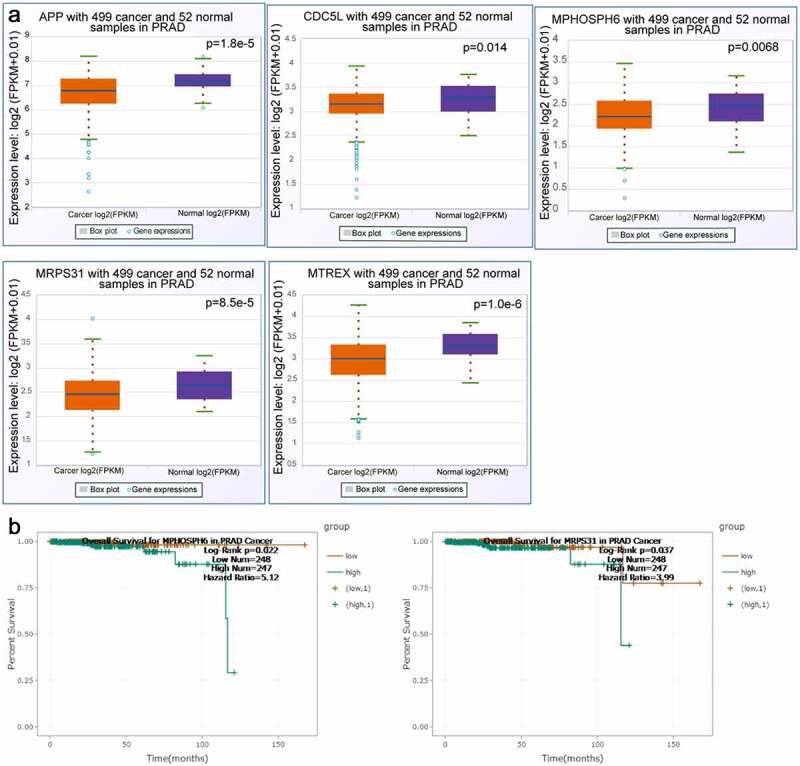
The correlation between selected gene expression levels and overall survival of PRAD patients. A, The expression level of selected genes in PRAD tissues and adjacent non-tumor tissues. B, The overall survival of human patients with PRAD in relation to high or low expression levels of MPHOSPH6 and MRPS31

The twenty-six node degree genes were analyzed using the starbase tool to determine whether there were any differences between the expression levels of PRAD and corresponding non-cancerous tissues. The expression levels of *APP, CDC5*L, *MPHOSPH6, MRPS31*, and *MTREX* in PRAD tissues were observed to be lower than those in corresponding non-cancerous tissues (Figure 4a). In addition, we found that the overall survival time of patients in the *MPHOSPH6* and *MRPS31* high expression groups was lower than that of patients in the respective low expression groups (Figure 4b). Furthermore, we analyzed the correlation of ZFC3H1 and *APP, CDC5*L, *MPHOSPH6, MRPS31*, and *MTREX* genes and examined the expression of these genes before and after ZFC3H1 knockdown. The results showed that transfection with ZFC3H1 siRNA could down-regulate *APP, CDC5*L, *MPHOSPH6, MRPS31*, and *MTREX mRNA* expression (Supplementary Figure 1 A-B).

### ZFC3H1 siRNA reduced PRAD cell migration and invasive capability

**Figure 5.**
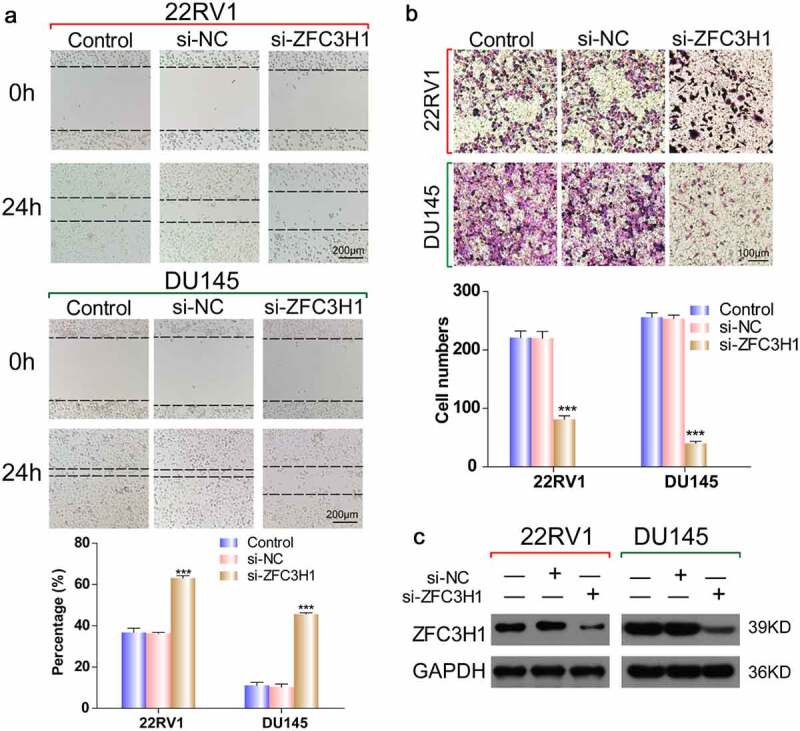
ZFC3H1 knockdown reduced PRAD cell invasive and migration capability. (a) Wound healing assay to determine the cell migration ability following transfection with the ZFC3H1 siRNA or negative siRNA. (b) Transwell assay showing the number of invaded cells following transfection with ZFC3H1 siRNA or negative siRNA. ***P < 0.001. (c) Western blotting showing the interference efficiency of ZFC3H1 siRNA

Increasing evidence suggests that ZFC3H1 is associated with cell mRNA translation. To verify whether silencing *ZFC3H1* could reduce the migration and invasion of PRAD cells, we transfected PRAD cells with ZFC3H1 siRNA or negative siRNA, then determined the interference efficiency of ZFC3H1 siRNA using western blotting. Western blotting showed that the interference effect is remarkable (Figure 5c). The wound healing assay determined that, compared with that of the negative control siRNA, *ZFC3H1* knockdown reduced PRAD cell motility and migration markedly (Figure 5a). The Transwell assay demonstrated significantly fewer invaded cells among the cells transfected with ZFC3H1 siRNA, compared with those among cells transfected with the negative control siRNA (Figure 5b). We also determined cell ability and cell apoptosis after ZFC3H1 siRNA treatment. The interference efficiency of ZFC3H1 siRNA was determined by RT-qPCR (Supplementary Figure 2 A). Knockdown of ZFC3H1 siRNA could reduce cell viability and increase the number of apoptotic cells in PRAD cells (Supplementary Figure 2 B-C). These results indicated that inhibiting ZFC3H1, suppressed PRAD cell migration and invasion significantly.

## Discussion

Given the high mortality and intractability of advanced PRAD, it is necessary to develop tools or markers to evaluate the prognosis of PRAD. The majority of PRAD initially depends on the androgen receptor (AR) pathway; therefore, treatments that target and inhibit androgen biosynthesis are widely used. While these therapies are initially effective, resistance and progression of PRAD often occurs, underscoring the need to develop new treatments [16]. ZFC3H1, as a central factor in the retention and degradation of polyadenylated RNA, is involved in the processing of a wide range of RNAs, and plays a crucial role in the degradation of nuclear RNAs [6–9]. It could be used in the future as diagnostic biomarkers and potential therapeutic targets for cervical SILs [25]. In addition, the depletion of ZFC3H1 resulted in a significant inhibition of translation [11]. Thus, the above studies show that ZFC3H1 has marked research value. To further understand the correlation between ZFC3H1 and the prognosis of PRAD, in the present study, we carried out bioinformatic analyses based on data from the TCGA database, and validated the results using human PRAD cells.

First, we found that ZFC3H1 expression levels in many cancers, including PRAD, were significantly lower compared with those in corresponding non-cancerous tissues. In addition, the survival time of patients with PRAD and/containing high ZFC3H1 expression groups was (was becomes ‘were’ if you use the word ‘and’) shorter than that in patients with low ZFC3H1 expression.

Second, we predicted 81 target genes of ZFC3H1 and overlapping target genes were examined via GO enrichment analyses to provide clues as to the functional roles of these genes in the biological processes of PRAD. A PPI network was constructed, and the most significant node degree genes (n = 26) were selected. We found that the expression levels of *APP, CDC5L, MPHOSPH6, MRPS31*, and *MTREX* in PRAD tissues were notably lower than those in corresponding non-cancerous tissues, and the patients in the *MPHOSPH6* and *MRPS31 *high-expression groups showed much shorter overall survival than patients in the respective low-expression groups. Furthermore, we further analyzed the correlation of ZFC3H1 and these five genes. Transfection with ZFC3H1 siRNA could down-regulate *APP, CDC5*L, *MPHOSPH6, MRPS31*, and *MTREX mRNA* expression. It is interesting that APP is the downstream signaling of ZFC3H1 because APP is a representative prognostic factor and regulated by the androgen receptor [26]. These findings support the hypothesis that ZFC3H1 plays an active role in PRAD development. To further demonstrate the effect of ZFC3H1 on the prognosis of PRAD, PRAD cells were transfected with an siRNA targeting ZFC3H1. Western blotting showed that the interference effect is remarkable. Subsequently, wound healing and Transwell invasion assays showed that *ZFC3H1* knockdown significantly inhibited PRAD cell migration and invasion abilities. CCK-8 and flow cytometry analysis showed that ZFC3H1 siRNA could reduce cell viability and increase the number of apoptotic cells in PRAD cells.

## Conclusions

In summary, we reported that ZFC3H1 is closely related to the migration and invasion ability of PRAD. It is also associated with cell viability and apoptosis in PRAD. ZFC3H1 or its regulated genes might provide new biomarkers for PRAD prognosis and provide a reference for the development of new therapies to treat PRAD. However, the low expression of ZFC3H1 in PRAD has not yet been explained and further studies are warranted.

## Supplementary Material

Supplemental Material

## Data Availability

The datasets used and/or analyzed during the current study are available from the corresponding author upon reasonable request.
