# Supplementary material for: Zinc finger C3H1 domain-containing protein (ZFC3H1) evaluates the prognosis and treatment of prostate adenocarcinoma (PRAD): A study based on TCGA data
Source: Bioengineered. 2021 Sep 13;12(1):5504–15. doi: 10.1080/21655979.2021.1965442 (PMC8806443; doi:10.1080/21655979.2021.1965442)
Supplement: Supplemental Material [file KBIE_A_1965442_SM7075.zip › suppl.pdf]

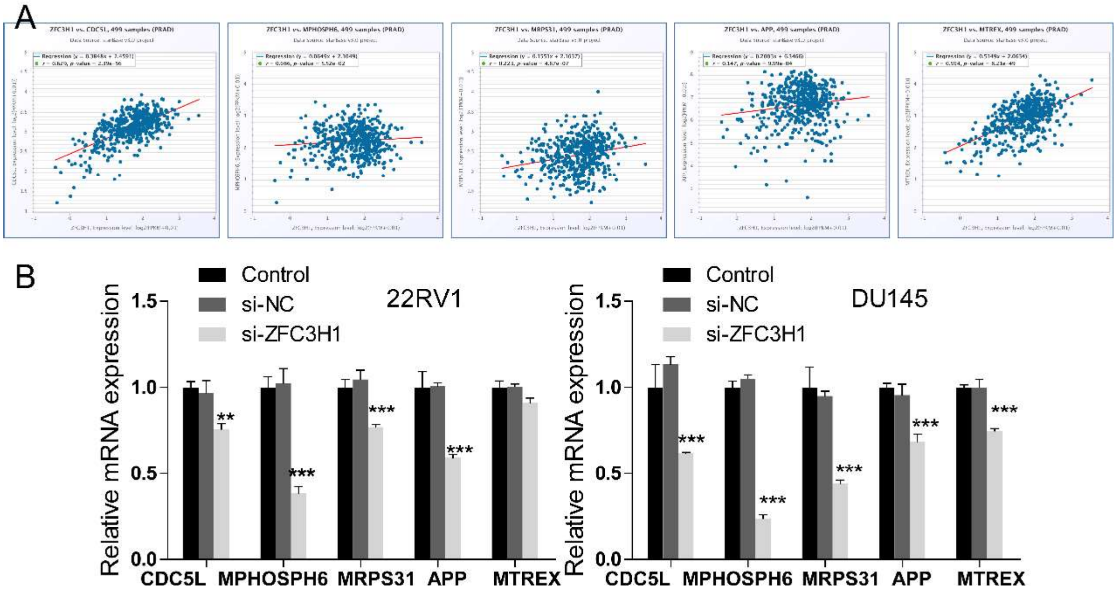

A. TCGA database alalyzed the correlation between ZFC3H1 and *APP*, *CDC5*L, *MPHOSPH6*, *MRPS31*, and *MTREX* gene. B.RT-qPCR detection *APP*, *CDC5*L, *MPHOSPH6*, *MRPS31*, and *MTREX* after transfection with ZFC3H1 siRNA. ***P < 0.001.

Supplementary Figure 2

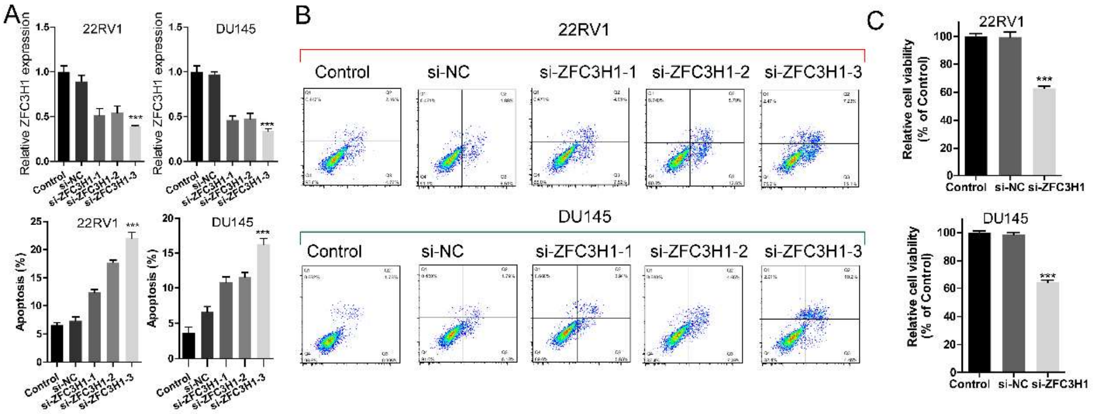

A. The expression of ZFC3H1 was determined by RT-qPCR analysis. ***P < 0.001.B. Flow cytometry analyzed cell apoptosis after it was transfected with ZFC3H1 siRNA. C. CCK-8 analyzed cell viability after transfection with or without ZFC3H1 siRNA. ***P < 0.001
